# Schmerzmanagement in der Inneren Medizin: Ergebnisse einer bundesweiten Erhebung zu Struktur- und Prozessdaten

**DOI:** 10.1007/s00482-021-00550-9

**Published:** 2021-04-20

**Authors:** M. I. Emons, T. H. Scheeper-von der Born, F. Petzke, V. Ellenrieder, L. Reinhardt, W. Meißner, J. Erlenwein

**Affiliations:** 1 Klinik für Anästhesiologie, Universitätsmedizin Göttingen, Robert-Koch-Str. 40, 37075 Göttingen, Deutschland; 2 Klinik für Gastroenterologie und gastrointestinale Onkologie, Universitätsmedizin Göttingen, Göttingen, Deutschland; 3 Klinik für Innere Medizin, Eichsfeld Klinikum, Heilbad Heiligenstadt, Deutschland; 4 Klinik für Anästhesiologie und Intensivmedizin, Universitätsklinikum Jena, Jena, Deutschland; 5 Klinik für Innere Medizin II, Abteilung Palliativmedizin, Universitätsklinikum Jena, Jena, Deutschland

**Keywords:** Akutschmerzmanagement, Akutschmerzdienst, Behandlungsstandards, Schmerzmedizin, Innere Medizin, Acute pain management, Acute pain service, Standards of care, Pain medicine, Internal medicine

## Abstract

**Hintergrund und Ziel der Arbeit:**

Seit Jahren werden Defizite der Qualität der Schmerztherapie im Krankenhaus beschrieben. Ziel der vorliegenden Untersuchung war es, Strukturen und Prozesse des Schmerzmanagements in internistischen Abteilungen darzustellen.

**Material und Methoden:**

Die Datenerfassung erfolgte mittels eines standardisierten Telefoninterviews (nichtuniversitäre Abteilungen); bei universitären Abteilungen separat mittels eines Onlinefragebogens (SurveyMonkey®).

**Ergebnisse:**

Daten von 139 nichtuniversitären Abteilungen (Rücklauf: 21 %) und 33 universitären Abteilungen (davon 21 vollständig beantwortete Fragebögen, Rücklauf 17 % bzw. 11 %) wurden erhoben. 441 von 619 kontaktierten nichtuniversitären Abteilungen lehnten die Teilnahme ausdrücklich ab, am häufigsten mit der Begründung, es bestünde kein Interesse am Thema Schmerzmanagement. In den 172 teilnehmenden Einrichtungen wurde Schmerz als eigenständiger Parameter in 89 % der nichtuniversitären Abteilungen (96 % universitär) regelmäßig während der Visite erfasst; schriftliche Behandlungsstandards zur Schmerztherapie lagen in 57 % der nichtuniversitären Abteilungen vor (54 % universitär). In 76 % der nichtuniversitären Krankenhäuser (100 % universitär) stand auch für die internistischen Patienten ein Akutschmerzdienst zur Mitbehandlung zur Verfügung, schriftliche Vereinbarungen zur Zusammenarbeit lagen bei 35 % vor (18 % universitär).

**Diskussion:**

Die berichtete Umsetzung des Schmerzmanagements war in den teilnehmenden Abteilungen gut. Gleichwohl sind die Ergebnisse bei niedriger Teilnahme und häufiger Ablehnung mit der Begründung einer fehlenden Relevanz nur eingeschränkt aussagekräftig. Die erfassten Daten reflektieren daher vermutlich eine erhebliche positive Selektion und sind nicht als repräsentativ für das Schmerzmanagement in der inneren Medizin zu werten.

## Abstract (en)

**Background, Objectives:**

Deficits in the quality of pain management in hospitals have been described for years. The aim of this study was to assess structures and processes of pain management in departments for internal medicine in German hospitals.

**Materials and methods:**

Data were collected using a standardized telephone interview (non-university hospital departments) on a randomized sample of hospitals (circa 1/3 of hospitals); all German departments of university hospitals were invited to participate and questioned separately using an online questionnaire (SurveyMonkey®).

**Results:**

Data from 139 non-university departments (response rate: 21%) and 33 university hospital departments (only 21 questionnaires were fully answered, response rate 17 and 11%, respectively) were collected. Of 619 non-university hospital departments contacted, 441 explicitly refused to participate in the survey, most often on the grounds that there was no interest in pain management. Pain was regularly recorded as an independent parameter during the medical visit in 89% of the non-university hospital and 96% of the university hospital departments; written standardized treatment protocols for pain therapy were available in 57% of the non-university hospital departments (54% university hospital departments). In 76% of the non-university hospitals departments (100% university hospital departments), an acute pain service was also available for patients of internal medicine departments for co-treatment. Written cooperation agreements were less common (35% non-university hospital departments, 18% university hospital departments).

**Conclusion:**

The described implementation of pain management was satisfying in the participating departments. However, in consideration of the low participation and high rejection of participation due to explicit disinterest in the topic, the results should be critically assessed and presumably characterized by a significant positive bias.

## Einleitung

Seit vielen Jahren werden durch nationale und internationale Erhebungen anhaltende Defizite in der Qualität der Schmerztherapie im Krankenhaus beschrieben [1, 16, 24, 25]. Diese Aussagen basieren jedoch meist auf Daten von operativen Patienten und nur wenige Untersuchungen beziehen sich auf die Versorgungssituation nichtoperativer Patienten. Die verfügbaren Erhebungen zeigen, dass Defizite in der Versorgungsqualität im konservativen Bereich denen der operativen Bereiche entsprechen [14, 20, 24]. Die Analyse von Teilaspekten lässt sogar darauf schließen, dass die Unter- und Fehlversorgung mit ausbleibender oder verzögerter Therapie von Schmerzen in den nichtoperativen Bereichen noch ausgeprägter ist [24]. Ein Bericht (Health Technology Assessment [HTA]) im Auftrag des Deutschen Instituts für Medizinische Dokumentation und Information und des Bundesgesundheitsministeriums bestätigt diese Einschätzung [20]. Versorgungsdefizite des Schmerzmanagements basieren mit auf fehlenden Regelungen zu Behandlungsabläufen und Verantwortlichkeit [10, 20, 26].

Erhebungen zur Akutschmerztherapie aus Sicht von Chirurgen oder Anästhesisten wurden bereits mehrfach durchgeführt [10, 21, 27, 30]. Informationen zu Strukturen und Prozessen des Schmerzmanagements in nichtoperativen Fachabteilungen lagen bisher nur aus zweiter Hand durch die Befragung anästhesiologischer Chefärzte vor und basierten nicht auf Angaben der Internisten selbst [13]. Bereits im Jahr 2016 sollten, analog zu den Vorbefragungen für die operative Medizin, in einem gemeinsamen Projekt zwischen Deutscher Gesellschaft für Innere Medizin e. V. (DGIM), der Deutschen Schmerzgesellschaft e. V. und unserer Arbeitsgruppe Struktur- und Prozessdaten erfasst werden. Hierzu wurden im Jahr 2016 alle als Chefärzte/leitende Ärzte registrierten Mitglieder der DGIM von ihrer Gesellschaft mit der Bitte um Teilnahme angeschrieben, inkl. einmaligen Reminders. Aufgrund des Rücklaufs von insgesamt 34 Fragebögen wurden Auswertung und Veröffentlichung verworfen.

Ziel des vorliegenden Projekts unserer Arbeitsgruppe war es, erneut aktuelle Struktur- und Prozessdaten des Schmerzmanagements in internistischen Fachabteilungen deutscher Krankenhäuser zu gewinnen. Aufgrund der Vorerfahrungen erfolgte die Datenerfassung jedoch mit adaptierter Erhebungsmethodik. Nichtuniversitäre Abteilungen wurden nach persönlicher Kontaktaufnahme per Telefoninterview befragt – aufgrund der großen Anzahl von Abteilungen allerdings anhand einer randomisiert ausgewählten Stichprobe. Da aus unserer Sicht aufgrund des meist höheren Spezialisierungsgrads und der damit verbundenen Heterogenität eine randomisierte Auswahl der universitären internistischen Abteilungen nicht sinnvoll erschien, wurden alle Einrichtungen persönlich angeschrieben und erhielten einen Link für eine Onlineerfassung der Fragen.

## Methodik und Materialien

### Kohorte und Randomisierung

Die Datenerhebung der Struktur- und Prozessdaten der nichtuniversitären Fachabteilungen für innere Medizin erfolgte prospektiv und randomisiert zwischen Juni und Oktober des Jahres 2018. Anhand des „Deutschen Krankenhausverzeichnisses“ (https://www.deutsches-krankenhaus-verzeichnis.de/, Stand 05.07.2017) wurden jeweils bundeslandspezifisch insgesamt 1317 nichtuniversitäre Krankenhäuser mit mindestens einer internistischen Fachabteilung ermittelt [4]. Ziel war es, für eine repräsentative Darstellung der Versorgungssituation hiervon ca. ein Drittel einzuschließen (450 Krankenhäuser). Die Auswahl der einzuschließenden Krankenhäuser erfolgte per Randomisierungsfunktion des Programms Excel (Microsoft Corporation, Redmond, USA, Version 2016) und konsekutiver Auswahl der in der entstehenden Reihung ersten 450 Kliniken. Anschließend wurden Kontaktdaten aller internistischen Einzelabteilungen der ausgewählten Krankenhäuser anhand einer Onlinerecherche erfasst (726 Abteilungen an 450 Krankenhäusern). 40 Abteilungen mussten ausgeschlossen werden, da die Angaben des Krankenhausverzeichnisses nicht mehr aktuell waren und zwischenzeitlich Schließungen oder Fusionierungen erfolgt waren, es sich um angeschlossene Praxen handelte oder um Krankenhäuser, für die in der Onlinerecherche keine zugehörige internistische Abteilung ermittelt werden konnte.

Nach Randomisierung und Onlinerecherche wurden für die einzuschließenden nichtuniversitären Krankenhäuser 686 eigenständige internistische Abteilungen ermittelt, und es erfolgte zunächst eine schriftliche Ankündigung der Studie sowie nachfolgend die telefonische Kontaktaufnahme.

### Datenerfassung

Die Kontaktaufnahme und Befragung der internistischen Abteilungen erfolgte von Juni 2018 bis Oktober 2018. Zur Verbesserung des Rücklaufs erfolgte die Kontaktaufnahme adaptiert an die jeweiligen Bundesländer unter Umgehung der Ferienzeiten. Per E‑Mail wurden die Chefärzte der Abteilungen jeweils ca. 2 Wochen vor Kontaktaufnahme angeschrieben und die Befragung angekündigt. Die telefonische Kontaktaufnahme erfolgte dann entsprechend im Abstand dieses Zeitfensters an Werktagen zu Geschäftszeiten, vorzugsweise vormittags. Abteilungen, die zu drei unterschiedlichen Zeitpunkten nicht telefonisch erreichbar waren, wurden nicht weiter kontaktiert.

### Erfassungsparameter

Für eine bessere Vergleichbarkeit mit bestehenden Daten für die operative Versorgung erfolgte die Erfassung mit einer reduzierten und in einigen Punkten angepassten Version des Akutschmerzzensus (2012), basierend auf den organisatorischen Eckpunkten der S3-Leitlinie „Behandlung akuter perioperativer und posttraumatischer Schmerzen“ ([22]; z. B. Regelungen von Verantwortlichkeiten, Rahmenvereinbarungen, Implementierung von Akutschmerzdiensten, -erfassung und -dokumentation, Konzepte zur Prophylaxe und Therapie). Die Datenerfassung erfolgte per standardisiertem Telefoninterview. Es wurden folgende Parameter erfasst:

1. **Allgemeine Angaben zum Krankenhaus** (Versorgungsstufe, Trägerschaft)
2. **Angaben zur Abteilung für innere Medizin** (Subspezialisierung, Bettenzahl, Position des Antwortenden)
3. **Schmerzerfassung und Dokumentation** (Frequenz der Schmerzerfassung, Effektivitätskontrolle nach Bedarfsanalgesie, Verfahren zur Schmerzerfassung, Art und Weise der Dokumentation)
4. **Behandlungsstandards** (Bedarfsmedikation, Handlungs- bzw. Interventionstrigger zur Bedarfsanalgesie, Verantwortlichkeit für die regelmäßige Überarbeitung der Standards, Einbindung nichtmedikamentöser Verfahren)
5. **Rahmenbedingungen des Schmerzmanagements und spezielle Versorgungsstrukturen** (Vorhandensein schriftlicher Vereinbarungen zur Zusammenarbeit in der Schmerztherapie, Einbindung von Schmerzdienst/schmerztherapeutischer Beratung)
6. **Qualitätssicherung und Zertifizierung** (regelmäßige Auswertung von Ergebnisdaten zur Darstellung der Ergebnisqualität, hausinterne Qualitätszirkel, Zertifizierung des Schmerzmanagements [Certkom, TÜV Rheinland], Implementierung des Expertenstandards „Schmerzmanagement in der Pflege“).

### Struktur- und Prozessdaten universitärer internistischer Fachabteilungen

In einem zweiten Schritt erfolgte die Datenerfassung bei universitären internistischen Fachabteilungen. Diese erfolgte per Onlinebefragung anhand desselben Fragenkatalogs. Die Erfassung der universitären Abteilungen erfolgte anhand Onlinerecherche bei allen internistischen Abteilungen der 34 Vollmitglieder des Verbands der Universitätsklinika Deutschlands e. V. (VUD) sowie drei Oldenburger Kliniken als assoziierte Mitglieder. Hierbei zeigte sich, dass ein universitäres Krankenhaus mit zwei Abteilungen sowie eine spezialisierte Fachabteilung bereits bei der Auflistung nichtuniversitärer Krankenhäuser selektiert wurden und diese bereits telefonisch befragt worden waren. Diese drei Abteilungen wurden nicht erneut kontaktiert und verblieben in der Kohorte der nichtuniversitären Abteilungen, da es sich nicht um Abteilungen eines langjährigen Universitätsklinikums handelte, sondern um Abteilungen universitärer Teilkrankenhäuser. Zwischen Januar und März 2019 wurden 195 universitäre internistische Abteilungen mittels persönlichen Anschreibens per Mail an die Direktoren kontaktiert und gebeten, die Onlinebefragung zu unterstützen. Es wurden zwei Reminder im Abstand von jeweils 3 Wochen versandt. Die Onlinebefragung erfolgte mittels der Onlinesoftware SurveyMonkey (SurveyMonkey, Inc, Palo Alto, Kalifornien, USA, 2019).

### Datenauswertung

Nach der Datenerfassung erfolgte eine anonymisierte Analyse in beiden Studienarmen. Die Darstellung der Ergebnisse erfolgte deskriptiv und getrennt zwischen nichtuniversitären und universitären Abteilungen. Ziel der Befragung war die Darstellung des Schmerzmanagements sowohl in nichtuniversitären als auch in universitären Abteilungen. Ein Vergleich zwischen nichtuniversitären und universitären Abteilungen war nicht beabsichtigt. Die Darstellung erfolgt deshalb getrennt. Prozentangaben wurden gerundet. Sofern nicht anders angegeben, beziehen sich die Angaben auf die eingeschlossenen Abteilungen. Die Auswertung der Daten erfolgte mit SPSS (IBM, Armonk, USA, Version 25).

## Ergebnisse

### Einschlüsse der nichtuniversitären Krankenhäuser

**Figure 1:**
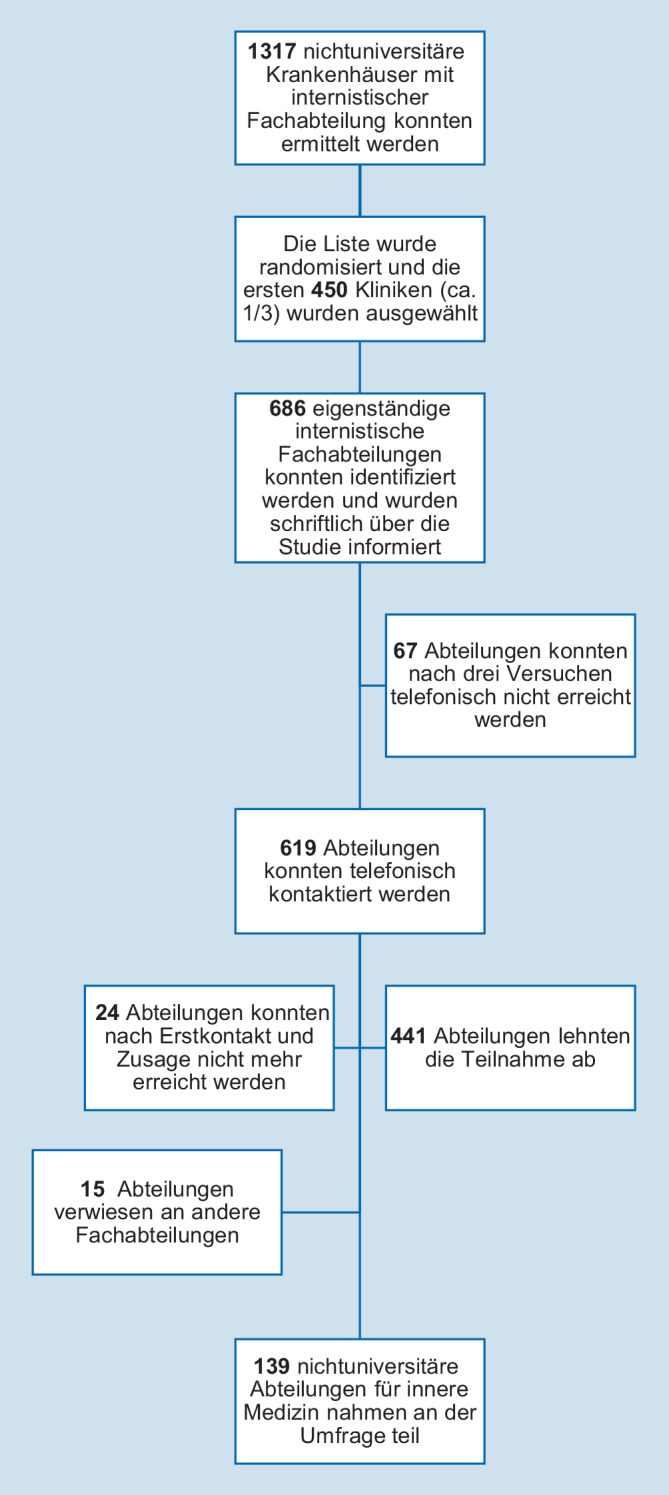

Von den 686 eigenständigen internistischen Abteilungen nahmen 139 an der Befragung teil (Abb. 1). Abteilungen, welche die Teilnahme aktiv ablehnten, begründeten dies am häufigsten ausdrücklich mit fehlendem Interesse am Thema Schmerzmanagement (*n* = 312), gefolgt von Zeit- (*n* = 119) und Personalmangel (*n* = 43). 8 Abteilungen gaben an, generell nicht an Befragungen teilzunehmen. Drei Abteilungen gaben als Begründung für die Nichtteilnahme an, keine Schmerzbehandlung zu betreiben (Mehrfachantworten möglich).

Die Interviewpartner waren in den meisten Fällen Oberärzte (40 %, *n* = 56) oder Chefärzte (32 %, *n* = 45), gefolgt von Weiterbildungsassistenten (20 %, *n* = 28) und Fachärzten (6 %, *n* = 8), sowie in je einem Fall eine Dokumentationsassistentin und eine Stationspsychologin (je 1 %). Die Strukturcharakteristika der teilnehmenden Abteilungen stellt Tab. 1 dar. Die Spezialisierungen der teilnehmenden Abteilungen sind in Tab. 2 zusammengefasst.

**Table 1:** 

Versorgungsstufe		Trägerschaft	
Grundversorgung	19 % (*n* = 27)	Öffentlich	35 % (*n* = 48)
Regel‑/Schwerpunktversorgung	52 % (*n* = 72)	Freigemeinnützig	39 % (*n* = 54)
Maximalversorgung	19 % (*n* = 27)	Privat	27 % (*n* = 37)
Fach- und/oder Belegklinik	8 % (*n* = 11)	–	–
Sonstiges	1 % (*n* = 2)	–	–

**Table 2:** 

Spezialisierung der Abteilung	Nichtuniversitäre Abteilungen^a^	Universitäre Abteilungen
Allgemeine innere Medizin	35 % (*n* = 48)	–
Gastroenterologie	27 % (*n* = 38)	18 % (*n* = 6)
Kardiologie	22 % (*n* = 30)	4 % (*n* = 1)
Geriatrie	23 % (*n* = 32)	–
Hämatologie-Onkologie	14 % (*n* = 20)	29 % (*n* = 8)
Pneumologie	8 % (*n* = 11)	11 % (*n* = 3)
Rheumatologie	4 % (*n* = 6)	11 % (*n* = 3)
Nephrologie	4 % (*n* = 6)	18 % (*n* = 5)
Endokrinologie	3 % (*n* = 4)	7 % (*n* = 2)
Angiologie	1 % (*n* = 1)	–
Sonstiges	–	21 % (*n* = 7^a^)

^a^Sonstiges: *n* = 4: Palliativmedizin, *n* = 2: internistische Intensivmedizin, *n* = 1: Gastroonkologie/Infektiologie

### Schmerzerfassung und Dokumentation (nichtuniversitäre Krankenhäuser)

Von den befragten nichtuniversitären Abteilungen gaben 89 % an, dass „Schmerz“ als eigenständiger Parameter während der ärztlichen Visite und/oder der Pflegevisite regelhaft erfasst wird (*n* = 124). In den meisten Abteilungen erfolgt dies laut Angaben mindestens einmal am Tag (38 %, *n* = 53; mind. 2 × täglich: 17 %, *n* = 24; „3 × täglich oder mindestens einmal pro Schicht“: 19 %, *n* = 26; „ab und zu“: 8 %, *n* = 11). In 76 % der Abteilungen (*n* = 105) wurde außerhalb der Routineerfassung (z. B. bei Patientenmeldung wegen Schmerz) die Schmerzintensität „meistens oder immer“ erhoben (*n* = 105; „ab und zu“: 19 %, *n* = 26; keine Schmerzmessung außerhalb der Routineerfassung: 6 %, *n* = 8). Zur Kontrolle nach einer medikamentösen Schmerztherapie wurde die Schmerzintensität nach Angaben der Befragten in etwa der Hälfte der Abteilungen „meistens oder immer“ erneut gemessen (50 %, *n* = 70; „ab und zu“: 25 %, *n* = 35; „nie“: 25 %, *n* = 34). Zur Schmerzerfassung wurde in der Mehrheit der Abteilungen ein standardisiertes Verfahren genutzt (90 %, *n* = 125).

Zur Effektivitätskontrolle der Schmerztherapie (Dokumentation durch die Stationspflege mind. 1 × täglich, Mehrfachantworten möglich) wurde am häufigsten der Analgetikaverbrauch dokumentiert und evaluiert (86 %, *n* = 120), gefolgt von der Schmerzintensität in Ruhe (81 %, *n* = 113) und von Nebenwirkungen (77 %, *n* = 107). Die Vigilanz bzw. der Sedierungsgrad wurde in 44 % der Abteilungen (*n* = 61) mindestens 1 × täglich dokumentiert.

### Behandlungsstandards und Verantwortlichkeiten (nichtuniversitäre Krankenhäuser)

Schriftliche Standards zur Behandlung von Schmerzen lagen zum Befragungszeitpunkt in 57 % (*n* = 79) vor (nur mündliche Vereinbarungen: 23 %, *n* = 32; keine Standards/Konzepte: 20 %, *n* = 28) und beinhalteten, wenn vorhanden, bis auf wenige Ausnahmen die Gabe einer Bedarfsmedikation (95 %, *n* = 75). Allerdings enthielten diese Standards nur in etwa der Hälfte der Fälle auch einen Interventionstrigger für die Gabe einer Bedarfsmedikation (53 %, *n* = 42). Nichtmedikamentöse Therapieverfahren waren in 47 % der Abteilungen mit schriftlichen Standards in diesen enthalten (*n* = 37).

Die Befragten gaben an, dass in 25 % der Abteilungen (*n* = 34) ausschließlich der Stationsarzt für das Schmerzmanagement verantwortlich war. In 40 % der Abteilungen (*n* = 56) stand zudem ein Akutschmerzdienst oder Schmerzdienst konsiliarisch zur Verfügung. In 35 % (*n* = 49) der Abteilungen gab es die Möglichkeit der aktiven Mitbehandlung durch einen Akutschmerzdienst, zum Beispiel durch Betreuung invasiver Verfahren. Zur Beratung bei komplexen schmerzmedizinischen bzw. palliativmedizinischen Fragestellungen stand in der überwiegenden Mehrzahl der Kliniken ein schmerzmedizinischer (87 %, *n* = 121) oder palliativmedizinischer Konsildienst (48 %, *n* = 67) zur Verfügung. Schriftliche Vereinbarungen zur Zusammenarbeit im Schmerzmanagement zwischen der schmerzmedizinischen Abteilung/dem Schmerzdienst und den internistischen Fachabteilungen lagen zum Zeitpunkt der Befragung in einem Drittel (35 %, *n* = 49) der Abteilungen vor.

### Qualitätsmanagement im Kontext Schmerztherapie (nichtuniversitäre Krankenhäuser)

Der Anteil der Abteilungen, in denen das Schmerzmanagement laut der Befragten zertifiziert war, lag bei 8 %. Gut die Hälfte gab an, dass ein solches Zertifikat geplant sei. In einem knappen Drittel war der Expertenstandard Schmerzmanagement in der Pflege implementiert. In 40 % der Kliniken gab es einen Qualitätszirkel, der sich mit der Akutschmerztherapie befasst und an dem sich in gut 2/3 der Fälle auch internistische Kollegen beteiligten (Tab. 3).

**Table 3:** 

	Nichtuniversitäre Abteilungen			Universitäre Abteilungen		
*–*	*Ja*	*Nein*	*Geplant*	*Ja*	*Nein*	*Geplant*
Schmerzmanagement des Hauses zertifiziert	8 %^a^ (*n* = 11)	86 %^a^ (*n* = 119)	7 % (*n* = 9)	50 %^b,c^ (*n* = 11)	32 %^b^ (*n* = 7)	18 % (*n* = 4)
Abteilung ist innerhalb dieses Zertifikats mit zertifiziert (oder dies wird angestrebt)	55 %^a^ (*n* = 6)	45 %^a^ (*n* = 5)	–	90 %^b,d^ (*n* = 9)	10 %^b^ (*n* = 1)	–
*–*	*Ja*	*Nein*	*Nicht bekannt*	*Ja*	*Nein*	*Nicht bekannt*
Daten zur Schmerztherapie werden mind. 1 ×/Jahr zur Qualitätssicherung ausgewertet	25 % (*n* = 34)	75 % (*n* = 105)	**–**	32 %^e^ (*n* = 6)	68 % (*n* = 13)	–
Expertenstandard „Schmerzmanagement in der Pflege“ in der Abteilung umgesetzt?	30 % (*n* = 42)	57 % (*n* = 79)	13 % (*n* = 18)	52 %^f^ (*n* = 11)	14 % (*n* = 3)	33 % (*n* = 7)
Regelmäßig tagender interner Qualitätszirkel, der sich auch mit der Akutschmerztherapie im Hause beschäftigt	40 % (*n* = 55)	36 % (*n* = 50)	25 % (*n* = 34)	24 %^f^ (*n* = 5)	24 % (*n* = 5)	52 % (*n* = 11)
Kollegen der Abteilung innere Medizin sind an diesem Zirkel regelmäßig beteiligt	67 % (*n* = 37)	33 % (*n* = 18)	–	40 % (*n* = 2)	60 % (*n* = 3)	–

^a^Zahlen durch Abgleich mit den Daten von Certkom/Deutsche Schmerzgesellschaft e. V. und TÜV Rheinland ermittelt

^b^Angaben der jeweiligen Abteilungen

^c^Fehlend: *n* = 11

^d^Fehlend: *n* = 23

^e^Fehlend: *n* = 14

^f^Fehlend: *n* = 12

### Schmerzmanagement in internistischen Abteilungen der universitären Medizin

Von den universitären Abteilungen nahmen 33 von 195 angeschriebenen Abteilungen (17 %) an der Onlinebefragung teil. Die vollständige Beantwortung der Fragen fiel im Verlauf des Fragebogens ab. Nur 21 der Abteilungen schlossen die Befragung auch ab (Rücklauf für komplettierte Fragebögen: 11 % der kontaktierten Abteilungen). Aufgrund der anonymen Onlinebefragung erfolgte keine Erfassung für Gründe einer ausbleibenden oder abgebrochenen Teilnahme. Die meisten universitären Abteilungen gehörten einem Klinikum mit über 1000 Betten an (73 %, *n* = 24; 700–999 Betten: 24 %, *n* = 8; 400–699 Betten: 3 %, *n* = 1). Beantwortet wurde der Fragebogen in den meisten Fällen von einem Oberarzt (44 %, *n* = 14), seltener vom Direktor/Chefarzt/Abteilungsleiter (19 %, *n* = 6), gefolgt von Assistenten in der Weiterbildung (16 %, *n* = 5) und Fachärzten (9 %, *n* = 3). In 12 % (*n* = 4) der Fälle wurde der Fragebogen von nichtärztlichen Kollegen beantwortet (jeweils 1 × genannt: „Bereichsleitung“, „Study Nurse“, „Gesundheits‑/Krankenpflegerin“, „Studienkoordinatorin“, fehlend: *n* = 1). Die Ergebnisse für die universitären Abteilungen sind in Tab. 2, 3, 4, 5 und 6 dargestellt.

**Table 4:** 

*Erfassung von „Schmerz“ während der Visite*^*a*^	
Ja	96 % (*n* = 26)
Nein	4 % (*n* = 1)
*Häufigkeit der Erfassung der Schmerzintensität*^*b*^	
1 ×/Tag	39 % (*n* = 10)
3 ×/Tag	31 % (*n* = 8)
2 ×/Tag	12 % (*n* = 3)
„Ab und zu“	12 % (*n* = 3)
Sonstiges	8 % (*n* = 2)
*Erfassung der Schmerzintensität durch ein standardisiertes Verfahren*^*b*^	
Ja	89 % (*n* = 23)
Nein	12 % (*n* = 3)
*Dokumentation der Schmerzerfassung*^*b*^	
In der Patientenkurve	73 % (*n* = 19)
Spezifische Akutschmerzdokumentation	4 % (*n* = 1)
Keine syst. Dokumentation	19 % (*n* = 5)
Sonstiges	4 % (*n* = 1)
*Erhebung der Schmerzintensität außerhalb Routineerfassung (z.* *B. Patientenmeldung)*^*b*^	
Meistens/immer	69 % (*n* = 18)
Ab und zu	27 % (*n* = 7)
Nie	4 % (*n* = 1)
*Erfassung der Schmerzintensität nach Verabreichen von Bedarfsmedikation*^*b*^	
Meistens/immer	58 % (*n* = 15)
Ab und zu	38 % (*n* = 10)
Nie	4 % (*n* = 1)

^a^Fehlend: *n* = 6

^b^Fehlend: *n* = 7

**Table 5:** 

*Vorliegen von Behandlungsstandards*^*a*^	
Schriftliche Standards	54 % (*n* = 14)
Mündliche Standards	27 % (*n* = 7)
Keine Standards	19 % (*n* = 5)
*Integration von Bedarfsmedikation in den Standard*^*b*^	
Ja	100 % (*n* = 14)
*Interventionstrigger für die Gabe einer Bedarfsmedikation*^*c*^	
Ja	87 % (*n* = 13)
Nein	13 % (*n* = 2)
*Integration von nichtmedikamentösen Therapien in den Standard*^*b*^	
Ja	36 % (*n* = 5)
Nein	64 % (*n* = 9)
*Verantwortlichkeit für die Überarbeitung der Standards*^*b*^	
Eigene Abteilung	36 % (*n* = 5)
Interdisziplinäre Arbeitsgruppe	57 % (*n* = 8)
Klinikweiter Schmerzbeauftragter	7 % (*n* = 1)
*Effektivitätskontrolle der Schmerztherapie (Mehrfachantworten möglich)*	
Schmerzstärke in Ruhe	70 % (*n* = 23)
Analgetikaverbrauch	52 % (*n* = 17)
Nebenwirkungen	52 % (*n* = 17)
Vigilanz	52 % (*n* = 17)

^a^Fehlend: *n* = 7

^b^Fehlend: *n* = 19

^c^Fehlend: *n* = 18

**Table 6:** 

*Verantwortlich für das Akutschmerzmanagement*^*a*^	
Stationsarzt und ASD (welcher zus. spezielle Verfahren betreut, z. B. PDK)	75 % (*n* = 18)
Stationsarzt und ASD (ohne weitere spezielle Verfahren)	13 % (*n* = 3)
Stationsarzt	12 % (*n* = 3)
*Unterstützung der Abteilung bei der Betreuung von Patienten mit Schmerzen*	
ASD^a^	100 % (*n* = 20)
Schmerzmedizinischer Konsildienst^b^	100 % (*n* = 23)
Palliativmedizinischer Konsildienst^c^	100 % (*n* = 23)
*Schriftliche Vereinbarung zur Zusammenarbeit mit der schmerzmedizinischen Abteilung/dem schmerzmedizinischen Bereich*^*d*^	
Ja	18 % (*n* = 4)
Nein	41 % (*n* = 9)
Beantwortende/r wusste nicht, ob Vereinbarungen vorlagen	41 % (*n* = 9)

*ASD* Akutschmerzdienst, *PDK* Periduralkatheter

^a^Fehlend: *n* = 8

^b^Fehlend: *n* = 13

^c^Fehlend: *n* = 9

^d^Fehlend: *n* = 10

Insgesamt wurde Schmerz als eigenständiger Parameter etwas häufiger erfasst als in den nichtuniversitären Abteilungen. Außerdem standen in den universitären Abteilungen in allen Fällen ein Akutschmerzdienst sowie ein palliativmedizinischer Konsildienst zur Verfügung. Auch bei den Angaben universitärer Häuser zum Qualitätsmanagement war der Anteil der Kliniken, die nach Einschätzung der Befragten Qualitätssicherung der Schmerztherapie betrieben, das Schmerzmanagement zertifiziert hatten bzw. ein Zertifikat anstrebten, wie bereits bei den nichtuniversitären Krankenhäusern hoch (Tab. 3).

## Diskussion

### Repräsentativität und Limitationen

Zur Erfassung von Strukturen und Prozessen des Schmerzmanagements in internistischen Abteilungen wurden alle internistischen Abteilungen einer randomisierten Stichprobe von ca. 1/3 der nichtuniversitären Kliniken in Deutschland sowie alle universitären internistischen Abteilungen kontaktiert und zur Teilnahme an der Erfassung aufgefordert. Die Ergebnisse der nichtuniversitären Kliniken stellen einen bundesweiten Querschnitt hinsichtlich ihrer Verteilung auf die einzelnen Bundesländer, die Krankenhausträger und Häuser unterschiedlicher Bettenanzahl dar [5]. Dennoch sind die Aussagen – wie es typisch für derartige Befragungserhebungen ist – in ihrer Repräsentativität limitiert. Dabei ist insbesondere ein erheblicher positiver Selektionsbias zu vermuten. Dies zeigt sich in einer großen Diskrepanz zwischen hohem Anteil an Ablehnungen bzw. ausdrücklichem Desinteresse am Thema Schmerzmanagement auf der einen und hoher Qualität der Struktur- und Prozessdaten der teilnehmenden Abteilungen auf der anderen Seite. Dies lässt vermuten, dass die Antworten zu einem großen Teil aus stärker engagierten Einrichtungen und von Kollegen, die für das Thema Schmerzmanagement sensibilisiert sind, stammten.

Nachdenklich macht nicht nur die Erfahrung aus der ersten Onlinebefragung (siehe oben), sondern auch der hohe Anteil an Abteilungen, die nicht nur – wie bei Befragungen mit teils vergleichbarer Rücklaufquote durchaus üblich – nicht teilnahmen, sondern mit Verweis auf fehlendes Interesse am Thema Schmerzmanagement die Teilnahme aktiv ablehnten.

Aufgrund der hohen Spezialisierung und damit verbundenen Heterogenität der universitären Abteilungen erfolgte die Befragung dort in einem zweiten Schritt an alle Abteilungen adressiert mittels Onlinefragebogen, um die Übersicht über die Versorgungslandschaft zu ergänzen. Während von den teilnehmenden nichtuniversitären Abteilungen alle Teilnehmenden auch die Befragung abschlossen, brachen 36 % der Kolleginnen und Kollegen der universitären Abteilungen die Onlinebefragung nach initialem Anklicken des Links im Verlauf der Beantwortung ab, sodass der Rücklauf für den vollständigen Fragebogen mit 11 % der kontaktierten Abteilungen auch im Vergleich zu anderen Befragungen zu diesem Thema sehr niedrig war [13, 15, 21, 29]. Aufgrund des höheren Rücklaufs und auch durch die Möglichkeit, Fragen im persönlichen Gespräch zu erläutern, muss für die Daten der nichtuniversitären Krankenhäuser von einer höheren Aussagekraft ausgegangen werden, sodass diese im Fokus der Darstellung standen. Wenn auch ein Vergleich zwischen nichtuniversitären und universitären Krankenhäusern nicht direkt beabsichtigt war, ist er aufgrund des unterschiedlichen Befragungsmodus zudem eingeschränkt.

Vor dem Hintergrund, dass Schmerzen oft das erste Symptom sind, das zur internistischen Untersuchung und/oder stationären Aufnahme führt, ist es schon verwunderlich, dass sowohl in den nichtuniversitären als auch in den universitären Kliniken nur wenig Interesse hinsichtlich des Themas Schmerzmanagement bestand. In dieser Befragung wurden leider nur die Fachabteilungen erfasst und nicht dezidiert gefragt, ob die Abteilungen auch Tumorpatienten behandeln. Es kann aber davon ausgegangen werden, dass eine Mehrheit der teilnehmenden Abteilungen und Kliniken sich auch mit onkologischen Patienten befasst und dass die Schmerztherapie auch unter diesem Aspekt eine wichtige Rolle spielen müsste.

## Struktur- und Prozessqualität

Alles in allem skizzierte sich bei den teilnehmenden Abteilungen eine gute Implementierung von Prozessen und Strukturen des Schmerzmanagements. Bei der Wahrnehmung und regelmäßigen Erfassung von Schmerz als eigenständigem Parameter zeigt sich ein deutlich besseres Bild im Vergleich zu der im Jahr 2012 im Rahmen des „Akutschmerzzensus“ durch die anästhesiologischen Kollegen für die nichtoperativen Bereiche gemachten Einschätzung [13]. Während dort 57 % der nichtoperativen Abteilungen angebenen hatten, Schmerzen nie standardmäßig zu erfassen, war dies in der vorliegenden Befragung nur in wenigen Abteilungen der Fall. Dennoch erfolgte in den meisten Abteilungen auch keine regelmäßige, mehrmals tägliche Erfassung (z. B. einmal pro Schicht) [13].

Diese positive Tendenz zeigte sich auch für die meisten anderen erfragten Prozess- und Strukturparameter zur Dokumentation, zu Behandlungsstandards und zu speziellen Versorgungsstrukturen des Schmerzmanagements wie beispielsweise dem Vorhandensein von Akutschmerzdiensten. Die Ergebnisse der hier vorgestellten Studie zeigen in den teilnehmenden Abteilungen ein annähernd ähnliches Bild der Implementierung von Strukturen und Prozessen des Schmerzmanagements, wie zuletzt in vielen Abteilungen der operativen Medizin [13].

Im Hinblick auf den Vergleich organisatorischer Anforderungen von Mitarbeitern der operativen und nichtoperativen Medizin an das Schmerzmanagement gibt es Hinweise, dass sich die organisatorischen Anforderungen gar nicht grundlegend unterscheiden [14]. Analog zur perioperativen Medizin konnten Einzelstudien zu prozedurenspezifischen Schmerzen in der konservativen Medizin zeigen, dass eine große Anzahl von Patienten postinterventionell unter mittelschweren bis schweren Schmerzen litten und diese durch ein strukturiertes Schmerzmanagement reduziert werden konnten [2, 7]. Allerdings lässt sich vermuten, dass sich die spezifischen Anforderungen an schmerzmedizinische Interventionen und Therapie zwischen den Patientenkollektiven unterscheiden und im Fall der internistischen Patienten über die in der operativen Medizin häufig im Vordergrund stehenden invasiven Analgesieverfahren (z. B. Periduralkatheter) oder opioidbasierten Therapieansätze hinausgehen [5, 12]. Bisher fehlen hierzu jedoch systematische Analysen und dementsprechend die Einbeziehung in Leitlinien. Perspektivisch ist hier die Einbeziehung nichtoperativer Aspekte, z. B. in entsprechende Leitlinien und Empfehlungen, wünschenswert. Auch wurden diese Aspekte in der Befragung nicht systematisch erhoben.

Neben dem zu vermutenden erheblichen Selektionsbias gibt es aber auch andere Gründe, die für die Notwendigkeit einer Verbesserung der Versorgungssituation sprechen könnten. So fokussierte das Projekt „Schmerzfreies Krankenhaus“, aus welchem die Zertifizierung Certkom „Qualifizierte Schmerztherapie“ entstanden ist, von Anbeginn nicht nur auf die operative Medizin, sondern bezog sich auf die nichtoperativen Bereiche. Auch hier zeigte sich, dass initial unter den am Projekt bzw. später der Zertifizierung teilnehmenden nichtoperativen Abteilungen eine im Verhältnis zur operativen Medizin schlechtere Ergebnisqualität vorlag. Es konnte jedoch über die Jahre in den neu zu zertifizierenden Kliniken bzw. in der Rezertifizierung tendenziell eine Verbesserung beobachtet werden ([23, 24], Folgedaten nicht veröffentlicht). Zum anderen hat inzwischen die Implementierung des „Expertenstandards Schmerzmanagement in der Pflege“ Verbreitung gefunden, und damit die Einbeziehung von Strukturen und Prozessen z. B. bzgl. des Schmerzassessments in der gesamten Klinik [5]. Immerhin ca. ein Drittel der Teilnehmenden berichtet, dass dieser in ihrer Abteilung implementiert sei. Auch zeichnet sich über die Jahre – auch in der Wahrnehmung aus Gesprächen mit Kolleginnen und Kollegen und der Autoren – die fortschreitende Entwicklung einer engeren innerklinischen Zusammenarbeit zwischen Schmerzdiensten und internistischen Abteilungen ab. Dieses Potenzial wurde auch in der im Jahr 2019 veröffentlichten Definition und Empfehlung der Deutschen Gesellschaft für Anästhesiologie und Intensivmedizin e. V. (Nürnberg) zu personellen und qualifikatorischen Anforderungen von Schmerzdiensten berücksichtigt [5, 11].

## Qualitätssicherung und „patient-reported outcomes“

Mit den Worten „If you can’t measure it, you can’t manage it“ betonte der österreichisch-US-amerikanische Ökonom Peter Ferdinand Drucker die Bedeutung der Definition von Erfolg und der Erfassung der Ergebnisqualität bei Managementprozessen und Qualitätsbeurteilung [8]. In unserer Befragung zeigten sich bei den Interviews große Unsicherheiten in Bezug auf die Qualitätssicherung und die Zertifizierung des Schmerzmanagements in den Abteilungen. Diese Fragen konnten im persönlichen Gespräch teils geklärt werden, zusätzlich konnte für die telefonisch befragten Abteilungen die tatsächliche Zahl der zertifizierten Abteilungen ermittelt werden. Etwa die Hälfte der befragten Abteilungen gab initial an, dass die Schmerztherapie im Haus zertifiziert sei (Tab. 3). Zum Zeitpunkt der Befragung (persönliche Anfrage beim TÜV Rheinland und bei der Deutschen Schmerzgesellschaft e. V. – seit dem Jahr 2017 Träger von Certkom; Stand Oktober 2018) waren in Deutschland und Österreich insgesamt 62 Krankenhäuser durch den TÜV Rheinland und 42 Krankenhäuser durch Certkom zertifiziert. Das entspricht bei 1942 deutschen Krankenhäusern (23.04.2020, DeStatis-Abfrage [3]) einem Anteil von 5 % aller deutschen Krankenhäuser. Insgesamt waren innerhalb dieser Zertifizierungen deutschlandweit lediglich 53 internistische Abteilungen enthalten, was die Angaben zur Zertifizierung des Schmerzmanagements fraglich macht, da es unwahrscheinlich ist, dass bei der Randomisierung ein derart hoher Anteil der zertifizierten Kliniken eingeschlossen wurde (TÜV Rheinland *n* = 17, Certkom e. V. *n* = 37, hier angegeben insgesamt *n* = 22). Auch der hohe Anteil universitärer Kliniken, die laut den Befragten ein zertifiziertes Schmerzmanagement hatten, ist fraglich bzw. lässt Zweifel an der Eindeutigkeit der Frage. Aufgrund der Anonymität der Umfrage konnten die Angaben für die nichtuniversitären Abteilungen nicht mit den Daten von Certkom e. V. und dem TÜV Rheinland abgeglichen werden. Vielmehr ist es wahrscheinlich, dass hier Unwissenheit vorlag oder diese sehr speziellen Zertifizierungen mit den Zertifizierungen von Organzentren, in denen auch oft Aspekte des Schmerzmanagements erfasst werden, verwechselt wurden.

Um den Qualitätsverbesserungsprozess des Schmerzmanagements zielgerichteter fortzusetzen, wäre eine regelmäßige Erfassung von „patient-reported outcomes“ (PRO), wie sie inzwischen in ca. 200 deutschen Krankenhäusern mit QUIPS (Qualitätsverbesserung in der postoperativen Schmerztherapie) erfolgt, auch in der internistischen Medizin sinnvoll. Hier besteht erhebliches Verbesserungs- bzw. Umsetzungspotenzial. Die Schwierigkeit der Befragung signalisiert aber auch, dass es wichtig ist, im breiten Kanon der internistischen Fächer die Abteilungen zu identifizieren, in denen ein solcher Aufwand sinnvoll und gewünscht ist. Unter den Teilnehmern erfolgt die Betrachtung von erhobenen Outcome-Parametern zur Qualitätssicherung des Schmerzmanagements nur in einem geringen Teil der Abteilungen. Das führt vermutlich zu „blinden Flecken“ und einer Unterschätzung der Relevanz, wie andere Veröffentlichungen vermuten lassen [6, 9, 14, 18–20, 24, 28, 31]. Auch in der operativen Medizin zeigte sich erst mit systemischen Erhebungen die Relevanz von Defizienten in der Ergebnisqualität des Schmerzmanagements [1, 16, 17]. Seit Kurzem existiert hierzu mit QUIKS (Qualitätsverbesserung im konservativen Schmerzmanagement) im Rahmen des QUIPS-Projekts auch ein Instrument zur systematischen Erfassung der Ergebnisqualität der Schmerzbehandlung in nichtoperativen Fachbereichen [9].

## Schlussfolgerung

Insgesamt zeigten die internistischen Adressaten häufig ausdrückliches Desinteresse am Thema Schmerzmanagement. Unter den internistischen Abteilungen, die sich zur Teilnahme bereiterklärten, zeichneten sich jedoch gute Strukturen und Prozesse des Schmerzmanagements ab. Die Repräsentativität dieses Ergebnisses ist jedoch aufgrund geringer Teilnahme und hoher Ablehnung des Themas Schmerzmanagement kritisch zu bewerten und vermutlich von einem erheblichen Positivbias geprägt. Nichtuniversitäre und universitäre Kliniken scheinen sich nicht grundlegend zu unterscheiden, wenn auch aufgrund des deutlich niedrigeren Anteils vollständig erfasster universitärer Abteilungen diese Einschätzung nur vage getroffen werden kann. Bzgl. organisatorischer Rahmenbedingungen (z. B. Implementierung von Behandlungsstandards, schriftliche Vereinbarungen zur Zusammenarbeit, regelmäßige Qualitätssicherung) besteht selbst bei teilnehmenden Kliniken die Möglichkeit eines Verbesserungspotenzials. Insbesondere der Erfassung von PRO sollte zur regelmäßigen Evaluation und Verbesserung der Qualität des Schmerzmanagements in der internistischen Medizin eine größere Bedeutung zukommen.

## Fazit für die Praxis

- In den Abteilungen für innere Medizin an universitären und nichtuniversitären Kliniken in Deutschland sollte dem Thema „Schmerzmanagement“ mehr Interesse entgegengebracht werden.
- In den teilnehmenden Kliniken zeichneten sich gute Strukturen und Prozesse ab.
- Die Repräsentativität dieses Ergebnisses ist jedoch aufgrund geringer Teilnahme und hoher Ablehnung des Themas Schmerzmanagement kritisch zu bewerten und vermutlich von einem erheblichen Positivbias geprägt.
- Verbesserungspotenzial zeigte sich in den teilnehmenden Kliniken vor allem bei den organisatorischen Rahmenbedingungen wie der Implementierung von Behandlungsstandards, schriftlichen Vereinbarungen und regelmäßiger Qualitätssicherung.
- Insbesondere der Erfassung von „patient-related outcomes“ (PRO) sollte zur regelmäßigen Evaluation und Verbesserung der Qualität des Schmerzmanagements in der internistischen Medizin eine größere Bedeutung zukommen.
